# Notch signaling pathway and heart development, congenital heart disease, and myocardial regeneration

**DOI:** 10.3389/fbioe.2025.1731605

**Published:** 2026-03-04

**Authors:** Yaping Liu, Xiaoxiao Wang, Xingyue Tao, Hao-Kun Zhang, Bingyan Liu, Yilin Li, Hang Wang, Huo-Min Luo, Hui-Lin Lv, Peifeng Li

**Affiliations:** 1 College of Food and Bioengineering, Zhengzhou University of Light Industry, Zhengzhou, China; 2 Henan Key Laboratory of Cold Chain Food Quality and Safety Control, Zhengzhou University of Light Industry, Zhengzhou, China; 3 Institute of Life and Health, Zhengzhou University of Light Industry, Zhengzhou, China

**Keywords:** cardiac development, congenital heart disease, myocardial regeneration, Notch signaling pathway, signal transduction

## Abstract

This review summarizes the critical role of the Notch signaling pathway in cardiac development, congenital heart disease, and myocardial regeneration. The Notch signaling pathway exerts a profound impact on cardiac health and disease progression by finely regulating the fate determination of cardiac progenitor cells, cardiac morphogenesis, and the proliferation and apoptosis of cardiomyocytes. The article also explores the research progress of the Notch signaling pathway as a potential therapeutic target and looks forward to future research directions.

## 1 Introduction

### 1.1 The central role of the Notch signaling pathway in the cardiovascular system

The pivotal role of the Notch signaling pathway in the cardiovascular system cannot be underestimated. This conserved ligand-receptor signaling pathway, first discovered in *Drosophila melanogaster* by Morgan et al., in 1917 (Zhou et al., 2022), has been confirmed to be widely present in most cellular organisms and is crucial for processes such as cell generation, apoptosis, proliferation, and stem cell self-renewal. The molecular components of the Notch signaling pathway include Notch receptors and their ligands, which, through interactions at the cell surface, trigger a series of intracellular signaling events that regulate the development and function of the cardiovascular system (Li et al., 2021).

The Notch signaling pathway plays an indispensable role in the development of the cardiovascular system. In the early stages of heart development, this pathway is involved in the formation of various cardiac structures. For instance, during the formation of the atrioventricular canal, activation of the Notch signaling pathway prompts a subset of endocardial cells to undergo epithelial-mesenchymal transformation (EMT), forming endocardial cushions that separate the atrioventricular canal and contribute to the construction of valve tissues. In ventricular development, Notch 1 is present in the endocardial cells at the base of the trabeculae and regulates myocardial cell proliferation by influencing the expression of related proteins such as BMP10, which is significant for early ventricular development. Numerous studies have shown that the Notch signaling pathway functions at multiple key stages of heart development and is an essential regulatory mechanism for normal heart development (Erhardt et al., 2021). The activity of the Notch signaling pathway directly affects cell fate decisions during heart development. This is particularly evident in valve formation, where Notch collaborates with local myocardial signals to guide vascular endothelial cells through endothelial-mesenchymal transformation (EMT), thereby forming the early primordia of heart valves (Wang Y. D. et al., 2021).

The Notch signaling pathway is also crucial during angiogenesis. It participates in the differentiation of vascular endothelial cells and the construction of the vascular network. Under normal conditions, the Notch signaling pathway maintains the balance of angiogenesis by regulating the expression and activity of factors such as vascular endothelial growth factor (VEGF). When the pathway is abnormal, angiogenesis becomes disordered; for example, abnormal activation of the Notch signaling pathway in tumor angiogenesis can promote abnormal growth of tumor blood vessels. In the cardiovascular system, it ensures the orderly progression of angiogenesis, maintains the normal structure and function of the vascular system, and provides sufficient blood supply to the heart and other tissues and organs (Akil et al., 2021).

**FIGURE 1:**
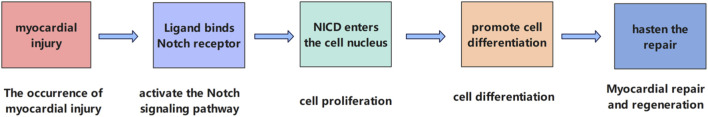
The role of Notch signaling pathway in myocardial injury repair.

In terms of myocardial injury repair, the Notch signaling pathway plays a key regulatory role. Studies have found that the Notch signaling pathway can negatively regulate the transformation of cardiac fibroblasts into myofibroblasts, reduce myocardial fibrosis, and thus promote myocardial repair. Following injuries such as myocardial infarction, activating the Notch signaling pathway can enhance the survival and regenerative capacity of myocardial cells, improving heart function. This indicates that the Notch signaling pathway holds a central regulatory position in the repair process after myocardial injury and provides an important potential target for myocardial repair treatments (Zhang et al., 2025) (Figure 1).

Given the central role of the Notch signaling pathway in the cardiovascular system, in-depth research into its molecular mechanisms and physiological functions is of great significance for understanding cardiac development, the occurrence of congenital heart diseases, and the regulatory mechanisms of myocardial regeneration. By unraveling the intricate regulatory network of the Notch signaling pathway, scientists are expected to develop novel therapeutic strategies targeting this pathway, opening up new avenues for the prevention and treatment of cardiovascular diseases. Future research will focus more on the mechanisms of action of the Notch signaling pathway in different cardiovascular diseases, as well as how to utilize the specific regulation of this pathway as a therapeutic target, with the aim of achieving the goals of precision medicine.

### 1.2 The importance of the association between the Notch signaling pathway and heart disease

The importance of the association between the Notch signaling pathway and heart disease lies in its central regulatory role in cardiac development, congenital heart disease, and myocardial regeneration. The Notch signaling pathway plays a crucial role in cardiac development, controlling the differentiation of cardiac progenitor cells, the formation of heart chambers, and the development of heart valves. Studies have shown that Notch signaling, by binding with its ligands (such as Jagged1, Dll4), activates downstream target genes (such as Hes1, Hey2), thereby influencing the differentiation and proliferation of cardiomyocytes. Abnormal expression or functional loss of Notch signaling can lead to cardiac developmental defects, such as ventricular septal defects and aortic valve malformations. Recent research has further revealed the interactive effects of Notch signaling with other developmental pathways (such as Wnt and BMP), providing new insights into the molecular mechanisms of congenital heart disease (Luxán et al., 2016; MacGrogan et al., 2010).

The occurrence of congenital heart disease (CHD) is closely related to abnormalities in the Notch signaling pathway. Genome-wide association studies (GWAS) and the application of gene editing technologies (such as CRISPR-Cas9) have revealed the association between mutations in multiple Notch pathway-related genes (such as NOTCH1, JAG1) and CHD. For example, NOTCH1 mutations are closely related to aortic valve disease and hypoplastic left heart syndrome, while JAG1 mutations are associated with cardiac defects related to Alagille syndrome. These findings clarify the pathological role of Notch signaling in CHD. Furthermore, they identify potential molecular targets for the early diagnosis and precision treatment of this disease (Evensen et al., 2025; Wang W. D. et al., 2025).

The role of the Notch signaling pathway in myocardial regeneration and repair has received extensive attention in recent years. Studies have shown that Notch signaling participates in the repair process following cardiac injury by regulating the proliferation and differentiation of cardiomyocytes. In myocardial infarction models, activation of Notch signaling can promote the proliferation of endogenous cardiomyocytes and improve cardiac function. Additionally, Notch signaling has been found to be closely related to the fate determination of cardiac stem cells, providing new research directions for stem cell-based regenerative medicine. However, overactivation of Notch signaling can also lead to fibrosis or arrhythmias, so its regulation requires a precise balance (Wang W. Y. et al., 2021; Kachanova et al., 2022).

Through studies on animal models, scientists have confirmed the key role of Notch signaling in developmental processes such as heart valve formation and ventricular septation. When Notch signaling is blocked or overactivated, normal cardiac development is disrupted, leading to various congenital heart diseases. For example, mutations in Notch2 and its ligand Jagged1 have been confirmed as one of the causes of Alagille syndrome, a genetic disease characterized by abnormal heart structure, particularly ventricular septal defects and right ventricular hypertrophy (Selvaggio et al., 2021) (Table 1).

**TABLE 1:** Tissue distribution of components of notch signalingin vascular system in vertebrates.

Component	Tissue/Organ	Distribution description	References
Notch1	Endothelial cells	Highly expressed in arterial endothelial cells	Liu et al. (2024a)
Notch2	Smooth muscle cells	Present in vascular smooth muscle cells	Radtke and Clevers (2005)
Jagged1	Endothelial cells	Found in arterial endothelial cells, involved in angiogenesis	Hasan and Fischer (2023)
Delta-like 1	Vascular progenitor cells	Expressed during vascular development	De Zoysa et al. (2022)
RBP-Jκ	Various tissues	Ubiquitously expressed in vascular tissues	Gradinaru and Ilie (2023)
Hes1	Endothelial cells	Regulated by Notch signaling in endothelial cells	Katoh and Katoh (2007)
DLL4	Endothelial cells	Specifically expressed in tip cells during angiogenesis	Lobov and Mikhailova (2018)
Notch3	Vascular smooth muscle cells	Vascular smooth muscle cells | Primarily expressed in vascular smooth muscle	Mao et al. (2021)
Notch4	Endothelial cells	Expressed in venous endothelial cells	O'Hare and Arboleda-Velasquez (2022)
CSL	Various tissues	Involved in transcriptional regulation of Notch target genes	Zeng et al. (2016)

## 2 The molecular biology basis of the Notch signaling pathway

### 2.1 The structure and function of the Notch receptor family

The Notch receptor family plays a central role in cell communication and fate determination, with members including four receptors, Notch1 to Notch4 (Cai and Dai, 2025). The structure of Notch receptor family members is highly conserved. For instance, the mammalian Notch receptor is a single-pass transmembrane protein, and its structure comprises three main parts: the extracellular, transmembrane, and intracellular regions. The extracellular region contains a large number (usually 29-36) of epidermal growth factor-like repeats (EGF-like repeats), which are structurally stabilized by specific disulfide bonds, providing the structural basis for ligand binding. Following this are the Lin12/Notch repeats (LNR), composed of three cysteine-rich domains, which are crucial for maintaining the structural stability of the extracellular region and regulating ligand binding affinity. The transmembrane region anchors the receptor firmly in the cell membrane, connecting the extracellular and intracellular parts. The intracellular region contains several important domains, such as the RAM domain that can bind to DNA-binding proteins (like CSL), the ANK repeat domain that mediates interaction with other proteins, the transcriptional activation domain (TAD) responsible for initiating downstream gene transcription, and the Pest domain rich in proline (P), glutamic acid (E), serine (S), and threonine (T), which is associated with receptor degradation (Sachan et al., 2024; Kopan and Ilagan, 2009).

In terms of ligand binding, Notch receptors exhibit high specificity. In mammals, there are five Notch ligands: Delta-like1, Delta-like3, Delta-like4, Jagged1, and Jagged2. Specific amino acid residues in the EGF-like repeats of the receptor’s extracellular region complementarily bind to the corresponding domains on the ligands, determining the specificity of the binding. Different EGF-like repeats have various functions in the binding process, with certain sequences determining the selectivity for Delta or Jagged ligands. The LNR domain affects the affinity of receptor-ligand binding through allosteric regulation. When the ligand binds to the receptor, the receptor conformation changes, exposing the cleavage site and initiating the subsequent signal transduction process (Palermo et al., 2014; Bigas and Espinosa, 2018).

Functionally, the Notch receptor family plays a key regulatory role in numerous biological processes such as cell fate determination, proliferation, differentiation, and apoptosis. When the Notch receptor binds to its ligand, it is sequentially cleaved by ADAM metalloproteases and γ-secretase, releasing the Notch intracellular domain (NICD). The NICD quickly translocates to the nucleus, binds to the transcription factor CSL, recruits co-activators such as MAML, forms a transcriptional activation complex, and initiates the transcription of downstream target genes (such as the Hes and Hey family genes). These target gene products further regulate cell behavior; for example, during embryonic development, the Notch signaling pathway is involved in the formation of various organs including the heart and blood vessels; in adult tissues, it maintains tissue homeostasis and regulates the balance of cell proliferation and differentiation. Abnormal Notch signaling pathway is closely associated with the development and progression of various diseases, such as cancer and cardiovascular diseases (Miyamoto and Rosenberg, 2011; Aster et al., 2017).

**FIGURE 2:**
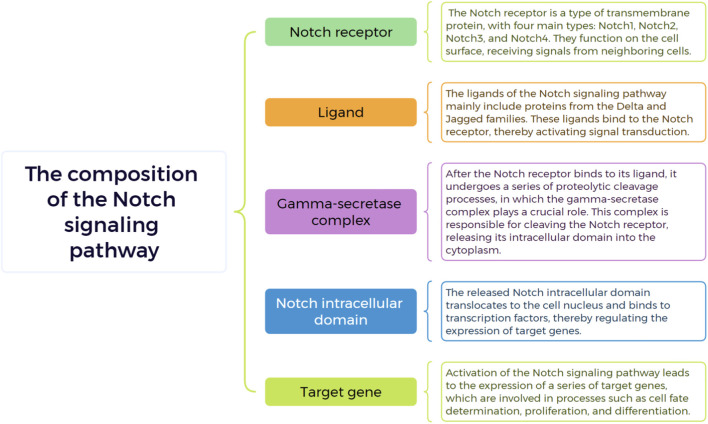
Key components of Notch signaling pathway.

It is noteworthy that the expression patterns of Notch receptor family members are closely related to heart diseases. For instance, variants of the NOTCH1 gene are closely associated with congenital heart diseases, and both gene deletions and gain-of-function mutations can lead to abnormal heart development, including atrioventricular septal defects and aortic valve stenosis (Roifman et al., 2021). Additionally, after myocardial infarction, although the expression of Notch1, Hes1, and Jagged-1 does not change significantly in the early phase, their expression increases in the chronically ischemic area, suggesting that the Notch signaling pathway may play an important role in cardiac repair and remodeling (Yang et al., 2011). These findings not only deepen our understanding of the role of the Notch signaling pathway in heart development and disease but also provide a theoretical basis for exploring new therapeutic strategies (Figure 2).

### 2.2 The role of the Notch signaling pathway in intercellular communication

**FIGURE 3:**
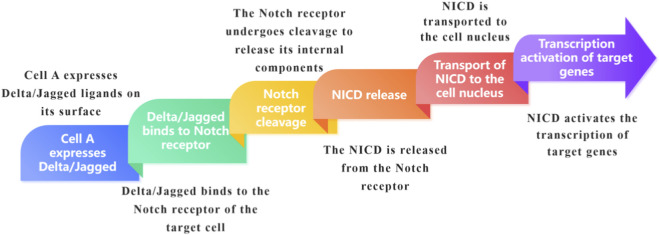
Brief overview of Notch signaling pathway.

The Notch signaling pathway plays a pivotal role in intercellular communication as a highly conserved mechanism of cell communication that is widely present in multicellular organisms. This communication primarily occurs through direct contact between Notch receptors on the surface of adjacent cells and their ligands. In mammals, there are four types of receptors: Notch1 - 4, and five types of ligands: Delta-like1, Delta-like3, Delta-like4, Jagged1, and Jagged2. When a ligand on the signaling cell binds to the Notch receptor on the receiving cell, it immediately triggers the signal transduction. This binding initiates a series of proteolytic events in the Notch receptor, first by the ADAM metalloprotease cutting the extracellular domain of the receptor, followed by a second cut by the γ-secretase complex in the transmembrane domain, releasing the Notch intracellular domain (NICD). The NICD then enters the nucleus and binds to the transcription factor CSL (also known as RBP-Jκ, CBF1, etc., in mammals), prompting CSL to switch from a transcriptional repressor complex to an activator complex. This, in turn, recruits co-activators such as Mastermind-like (MAML) proteins, activating the transcription of target genes like Hes and Hey, ultimately regulating cell fate, differentiation, and proliferation. For example, during embryonic development, communication through the Notch signaling pathway between neighboring cells can determine the direction of cell differentiation into different tissue cells, which is crucial for organ formation and morphogenesis. In adult tissues, it maintains intercellular homeostasis and regulates the balance of cell proliferation and differentiation (Wang F. et al., 2020; Tsaouli et al., 2020; Ebrahimi, 2023) (Figure 3).

A landmark study revealed that during mammalian heart development, cardiomyocytes can extend tunneling nanotube-like structures up to several micrometers in length. These structures directly penetrate the noncellular “cardiac jelly” layer and physically connect distant endocardial cells. Acting as “biological cables,” these nanotubes specifically transport membrane-bound Notch ligands (such as Jagged1), enabling precise signal transmission across tissue layers. Disruption of these structures leads to a loss of Notch signaling pathway in the endocardium and severe defects in cardiac trabeculation, demonstrating that this is an essential and active ligand-delivery mechanism (Miao et al., 2025).

Studies have found that various components of the Notch pathway, including transmembrane ligands, the extracellular domains of receptors, and even the activated intracellular domains, can be encapsulated into extracellular vesicles such as exosomes. These vesicles circulate through bodily fluids and are taken up by distant cells, thereby activating or inhibiting Notch signaling in target cells without the need for direct cell contact. For example, exosomes derived from tumor cells can influence the distant microenvironment by delivering Notch ligands, providing a novel mechanism for the role of Notch signaling pathway in cancer metastasis and systemic regulation (Yang et al., 2021).

This characteristic of the Notch signaling pathway has a wide range of applications in various physiological processes. For instance, during heart development, the Notch signaling pathway ensures the normal formation of heart structure by regulating the proliferation, differentiation, and apoptosis of cardiomyocytes (Piñeiro-Sabarís et al., 2024). Among cardiomyocytes, the activation of Notch signaling pathway promotes the determination of cell fate, maintaining the orderly progression of heart development.

## 3 Notch signaling pathway in cardiac pathophysiology and repair

### 3.1 The role of the Notch signaling pathway in heart development

#### 3.1.1 The regulation of heart development by the Notch signaling pathway

**FIGURE 4:**
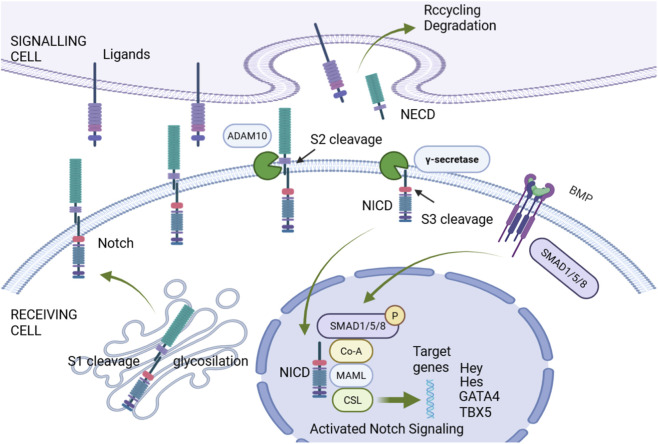
Notch signaling pathway.

Cardiac development is a complex and precise process that begins in the early embryo and involves the differentiation and cooperation of multiple cell lineages. During the third week of embryonic development, the cardiac primordia begin to form, and subsequently, through steps such as cardiac tube formation, looping, and septation, it gradually develops into a four-chambered heart. This process is regulated by various signaling pathways (such as Wnt, Notch, BMP, etc.) and transcription factors (such as NKX2-5, TBX5, GATA4, etc.). Abnormalities in cardiac development can lead to the occurrence of congenital heart disease, making the study of the molecular mechanisms of cardiac development crucial for understanding the etiology of congenital heart disease (Figure 4) (Zhao, 2024).

The regulatory role of the Notch signaling pathway in heart development is extensive and critical, spanning all stages of cardiac development. In the early stages of heart development, the Notch signaling pathway is involved in determining the fate of cardiac progenitor cells. Studies have shown that in the developing embryonic heart of mice, Notch1 is highly expressed in cardiac progenitor cells, where its activity maintains the undifferentiated state of these cells, inhibiting premature cardiomyocyte differentiation and ensuring an adequate pool of progenitor cells for heart development. During the stage of cardiac morphogenesis, the Notch signaling pathway regulates the formation of multiple cardiac structures. For instance, during the formation of the atrioventricular canal, the activation of the Notch signaling pathway prompts some endocardial cells to undergo epithelial-mesenchymal transformation (EMT), leading to the formation of endocardial cushions, which is crucial for the separation of the atrioventricular canal and the construction of valve tissues (Hikspoors et al., 2025). In the process of ventricular development, Notch1 is present in the endocardial cells at the base of the trabeculae and regulates cardiomyocyte proliferation and trabeculation formation by influencing the expression of related proteins such as BMP10 (Del Gaudio et al., 2022). Additionally, the Notch signaling pathway also plays a significant role in the development of the cardiac conduction system. During the formation of the sinoatrial node, the pacemaker of the heart, Notch1 signaling is recruited in the endocardium and promotes the formation of the venous sinus valve and the sinoatrial node by upregulating Wnt signaling (Collu et al., 2014).

In the later stages of heart development, the reactivation of Notch signaling pathway is considered an adaptive response of the adult heart to pathological injury. It is noteworthy that the role of Notch signaling pathway in heart development is not isolated; it interacts complexly with other signaling pathways such as Wnt/β-catenin, BMP, and FGF signaling pathways, collectively shaping the final morphology of the heart (Wang Y. D. et al., 2020). However, the specific mechanisms of Notch signaling pathway in the later stages of heart development and its relationship with heart diseases still require further research for clarification.

#### 3.1.2 Critical aspects of Notch function in cardiac morphogenesis

The Notch signaling pathway plays a crucial role in the process of cardiac differentiation and proliferation. This pathway regulates the fate determination of cardiac precursor cells, affecting the proliferation and differentiation of cardiomyocytes. In the early stages of embryonic development, specific Notch receptors and ligands exhibit dynamic expression patterns in cardiac precursor cells. Taking Notch1 as an example, it is highly active in cardiac progenitor cells and interacts with downstream target genes to inhibit the premature expression of cardiomyocyte-specific genes, thereby maintaining the undifferentiated stemness state of cardiac progenitor cells and reserving sufficient cellular resources for the precise differentiation of subsequent cardiac compartments. Studies have shown that when Notch1 signaling is artificially blocked, cardiac progenitor cells differentiate abnormally early, leading to disorders in cell lineage allocation during heart development and affecting the formation of normal heart structure (Gomez et al., 2021).

As development progresses, the Notch signal is also involved in the differentiation choices of different cell types during the specific differentiation stages of cardiac structures. For instance, in the atrioventricular canal region, the activation of the Notch signaling pathway guides some endocardial cells to transform into mesenchymal cells, a process known as epithelial-mesenchymal transition (EMT), which is crucial for the formation of endocardial cushions and the subsequent development of atrioventricular valves (McIntyre et al., 2020).

In terms of cardiac cell proliferation, the Notch signal also plays an indispensable role. In the early stages of ventricular development, Notch1 in the endocardial cells at the base of the trabeculae regulates the expression of a series of growth factors and signaling molecules, such as upregulating bone morphogenetic protein 10 (BMP10), to promote cardiomyocyte proliferation, which contributes to the thickening of the ventricular wall and the normal development of the trabecular structure. If Notch signaling pathway is impaired at this stage, the reduced rate of cardiomyocyte proliferation can lead to ventricular hypoplasia and affect heart function (Zhao L. et al., 2019). Moreover, when the adult heart responds to injury, the Notch signaling pathway can be reactivated, stimulating cardiomyocytes to re-enter the cell cycle, thus promoting cardiomyocyte proliferation to some extent and participating in the heart’s repair process (Acar et al., 2021).

The regulatory mechanism of the Notch signaling pathway also demonstrates its importance in the proliferation and differentiation of dental follicle cells (rDFCs) (Zheng et al., 2022). Notch-1 is considered a specific marker of DFCs, and the activation of its signaling pathway can inhibit apoptosis and promote cell proliferation (Kozyrev et al., 2020). Through overexpression or silencing of Notch1, researchers have found that the activation of Notch1 signaling can reduce the apoptotic effect, inhibit the expression of the p53 gene, and thereby promote cell survival and proliferation (High and Epstein, 2008). These findings emphasize the promotional role of Notch signaling in the proliferation and differentiation processes of cardiac cells, providing new insights into understanding heart development and diseases.

#### 3.1.3 The relationship between Notch signaling pathway and cardiac growth

The Notch signaling pathway plays a crucial role in the process of cardiac growth, with its regulation of heart size and morphology directly impacting the functional maturation and health status of the heart. In the early stages of embryonic development, the activity level of Notch signaling can determine the number and proliferation rate of cardiac progenitor cells, thereby influencing the initial size of the heart (Ye et al., 2023). This process involves the interaction of Notch signaling with other key developmental signaling pathways, such as the Wnt signaling pathway, which together determine the fate of cardiac progenitor cells and the ultimate morphology of the heart organ (Miyamoto et al., 2021).

### 3.2 The Notch signaling pathway and congenital heart disease

Congenital Heart Disease (CHD) is one of the most common birth defects, affecting approximately 1% of live births. Its etiology is complex and involves the interplay of genetic factors (such as chromosomal abnormalities, single gene mutations) and environmental factors (such as maternal infections, drug exposure). In recent years, with the development of genomics and epigenetics technologies, researchers have identified multiple genes and regulatory regions associated with CHD, such as NKX2-5, TBX5, and NOTCH1. These findings not only reveal the molecular basis of CHD but also provide potential targets for early diagnosis and intervention (Pierpont et al., 2018).

#### 3.2.1 The association between Notch signaling pathway abnormalities and congenital heart disease

There is a close association between abnormalities in the Notch signaling pathway and congenital heart disease (CHD). The Notch signaling pathway plays a crucial role at multiple stages of cardiac development, including the formation of the heart tube, atrioventricular canal, valves, outflow tract, coronary arteries, and the atrioventricular node (Liu K. et al., 2024). During cardiac development, Notch signaling pathway is essential for the formation of various cardiac structures and the determination of cell fate. Studies have shown that mutations in the Notch1 gene can lead to abnormal function of the encoded protein, thereby affecting cell differentiation and proliferation during heart development (Peng, 2023). For instance, in the development of heart valves, the normal activation of the Notch signaling pathway is vital for the differentiation of endocardial cushion cells and the formation of valve structures. If the Notch signaling pathway is abnormal, such as the single nucleotide polymorphisms (SNPs) in genes related to the Notch signaling pathway observed in some patients with congenital heart disease, it can disrupt this process, leading to valvular malformations and congenital valve diseases (Kefalos et al., 2019). Additionally, during the formation of the atrioventricular septum, the Notch signaling pathway is involved in regulating the proliferation and migration of myocardial cells; abnormal Notch signaling pathway can disrupt this balance, resulting in the occurrence of septal defects in congenital heart disease (Kodo et al., 2021). Furthermore, research suggests that abnormal Notch signaling pathway may also affect the development of the cardiac conduction system, increasing the risk of congenital arrhythmia heart diseases (Zakariyah et al., 2018).

Abnormalities in the Notch signaling pathway may also indirectly promote the occurrence of congenital heart disease by affecting the proliferation, differentiation, and apoptosis of cardiac cells. In summary, abnormal Notch signaling pathway plays a significant role in the pathogenesis of various types of congenital heart disease. Therefore, a thorough understanding of the mechanisms of the Notch signaling pathway in congenital heart disease is of great importance for the development of targeted therapeutic strategies.

#### 3.2.2 Research on the role of Notch signaling pathway in the pathogenesis of congenital heart disease

In the study of the pathogenesis of congenital heart disease, the Notch signaling pathway plays a crucial role. Abnormalities in this signaling pathway are closely associated with a variety of cardiac defects, including anomalies in the heart loop, atrioventricular canal, valves, outflow tract, coronary arteries, and atrioventricular node (Stanley et al., 2024).

The Notch signaling pathway is a highly conserved pathway that regulates the growth and development of organisms through intercellular interactions. During embryogenesis, the Notch signaling pathway is involved in programming multiple processes from cardiac structure to cardiac electrophysiological characteristics, playing an important role in the development of the heart and the formation of the conduction system (Xiang et al., 2025). During embryonic development, the activation of Notch signaling pathway regulates the fate determination, proliferation, apoptosis, and migration of cardiac precursor cells. However, when Notch signaling pathway is dysregulated, the delicate balance in the process of cardiac development is disrupted, leading to the occurrence of congenital heart disease.

In recent years, extensive research has been dedicated to exploring the potential role of distant non-coding regulatory genomic regions in organ development and disease. In particular, genomewide association studies (GWAS) have been used to identify disease-risk variants located at a distance, especially those associated with valvular diseases (Luna-Zurita et al., 2023). The formation of the endocardial cushion and the subsequent epithelial-mesenchymal transition (EMT) constitute the foundation for the development of the atrioventricular valves and septa. Notch signaling pathway directly regulates the expression of key EMT transcription factors, such as Snai1. Deficiency in Jagged1-Notch signaling impairs the migration and proliferation of endocardial cushion mesenchymal cells, which represents one of the core mechanisms leading to atrioventricular septal defects (AVSD) (Metrich et al., 2015). In aortic valve development, Notch1 activity within endothelial cells maintains the normal structure and function of valve leaflets by inhibiting osteogenic-like differentiation. The loss of its function serves as an initiating factor for bicuspid aortic valve (BAV) and its subsequent calcification (Adhicary et al., 2023).

During the networking and compaction of ventricular myocardial trabeculae, Notch signaling acts as a critical “brake.” Specifically, Notch signaling originating from the endocardium precisely modulates the expression of paracrine factors (such as Nrg1 and Bmp10), thereby controlling the proliferation rate of outer layer myocardial cells. Excessively strong signaling can inhibit myocardial cell proliferation, potentially leading to ventricular wall thinning; conversely, insufficient signaling may result in excessive trabecular growth and compaction defects (Wang et al., 2013). On the other hand, cardiac neural crest cells (cNCCs) are indispensable for the proper septation of the outflow tract and the aortopulmonary septum. Research confirms that Notch signaling pathway activity, both within the cNCCs themselves and in the pharyngeal arch arterial endothelial cells they target, is crucial for the survival, migration, and differentiation of cNCCs. Experiments using conditional knockout mouse models have demonstrated that disruption of this pathway can lead to conotruncal malformations such as persistent truncus arteriosus and tetralogy of Fallot (Erhardt et al., 2021).

Recently, with advancements in molecular biology techniques and research using animal models, the specific mechanisms of the Notch signaling pathway in the pathogenesis of congenital heart disease have become increasingly clear. Studies have shown that the Notch signaling pathway interacts with the PI3K/Akt and NF-κB signaling pathways, jointly participating in myocardial protective mechanisms (Wang et al., 2024).

#### 3.2.3 The potential of Notch signaling pathway as a therapeutic target for congenital heart disease

Delving into the association between the Notch signaling pathway and congenital heart disease reveals that Notch signaling pathway abnormalities often accompany abnormal development of cardiac structures, such as ventricular septal defects, patent ductus arteriosus, and other common congenital heart diseases (Theis et al., 2015). These findings suggest that by regulating the Notch signaling pathway, it may be possible to correct errors in cardiac development, thereby preventing or treating congenital heart disease.

Given the important role of the Notch signaling pathway in cardiac diseases, its potential as a therapeutic target is broad. Currently, researchers are developing drugs targeting the Notch signaling pathway, aiming to achieve effective intervention in congenital heart disease through precise regulation of the pathway’s activity (Zhang Y. et al., 2022).

### 3.3 Notch signaling pathway and myocardial regeneration

Myocardial regeneration is one of the hot topics in cardiovascular research, aiming to repair myocardial damage caused by myocardial infarction or cardiomyopathy. The regenerative capacity of adult mammalian cardiomyocytes is limited, leading researchers to explore various strategies, including stem cell transplantation, gene editing, and epigenetic regulation. In recent years, research on induced pluripotent stem cells (iPSCs) and cardiac progenitor cells has provided new hope for myocardial regeneration. Additionally, activating endogenous cardiomyocyte proliferation or reprogramming fibroblasts into cardiomyocytes has become a potential therapeutic direction (Tzahor and Poss, 2017).

#### 3.3.1 Regulation of Notch signaling pathway in myocardial cell apoptosis

The Notch signaling pathway plays a complex and critical role in the regulation of myocardial cell apoptosis. Under normal physiological conditions, the Notch signaling pathway maintains a certain level of activity, which inhibits myocardial cell apoptosis. Studies have shown that in a mouse model of myocardial ischemia-reperfusion injury, enhancing the activity of the Notch signaling pathway can significantly reduce the rate of myocardial cell apoptosis and improve cardiac function (Fang et al., 2019). However, under certain pathological conditions, the regulatory pattern of Notch signaling pathway may change. When the myocardium is subjected to sustained oxidative stress or inflammatory stimulation, the Notch signaling pathway may be overactivated or abnormally suppressed. Overactivation of Notch signaling pathway can lead to a series of signaling disorders, resulting in mitochondrial dysfunction in myocardial cells, the release of cytochrome C, and subsequently activating the caspase cascade reaction, promoting myocardial cell apoptosis (Zhou et al., 2018; Xu et al., 2024). Conversely, inhibiting the Notch signaling pathway may also disrupt the normal intracellular homeostasis, making myocardial cells more sensitive to apoptotic stimuli (Xu, 2021).

Additionally, there is an interaction between the Notch1 signaling pathway and the Keap1-Nrf2 signaling pathway, which together participate in the protective effects against myocardial ischemia-reperfusion injury. Under hypoxia-reoxygenation conditions, the synergistic action of the Notch1 and Keap1-Nrf2 signaling pathways significantly increases the survival rate of myocardial cells, inhibits myocardial cell apoptosis, reduces the formation of reactive oxygen species (ROS), and enhances antioxidant capacity. These findings suggest that the interaction of the Notch1-Nrf2 signaling pathway plays a protective role in the myocardium by reducing the generation of ROS (Zhou et al., 2020).

#### 3.3.2 The role of Notch signaling pathway in the process of myocardial regeneration

The Notch signaling pathway plays a crucial role in the process of myocardial regeneration. Taking zebrafish as an example, this model organism exhibits complete heart regeneration capabilities, making it an ideal model for exploring the molecular mechanisms of myocardial cell regeneration. Following ventricular injury in zebrafish, the Notch1 signaling pathway is significantly activated in the endocardium, promoting heart regeneration (Xiao et al., 2023).

The specific mechanisms of action of the Notch signaling pathway in myocardial regeneration involve multiple aspects. On one hand, the activation of Notch signaling pathway can promote myocardial cell proliferation events, primarily achieved by inducing the expression of bone morphogenetic protein 10 (BMP10) in adjacent myocardial cells (Mensah and Gowher, 2024). Further research reveals that the Notch signaling pathway works in concert with other developmental regulation pathways such as Wnt, Hedgehog, and bone morphogenetic protein/TGF-β, collectively determining the differentiation state and regenerative capacity of myocardial stem cells (Sottoriva and Pajcini, 2021). In particular, after cardiac injury, the activation of Notch signaling pathway can stimulate the proliferation of myocardial stem cells and promote the repair of cardiac tissue.

#### 3.3.3 The interaction between the Notch signaling pathway and macrophages in myocardial regeneration

During the process of myocardial regeneration, there exists a complex interaction between macrophages and the Notch signaling pathway. Following myocardial ischemia, macrophages undergo a transition from pro-inflammatory M1 to anti-inflammatory M2 phenotype, which is crucial for myocardial repair. The Notch signaling pathway plays a key role in regulating the phenotypic conversion of macrophages, and changes in its activity directly affect the proportion of M1 and M2 macrophages, thereby influencing the efficiency of myocardial regeneration.

The interaction between the Notch signaling pathway and macrophages is also influenced by a variety of signaling pathways, forming a complex regulatory network. For example, after myocardial ischemic injury, Notch signaling pathway interacts with interferon regulatory factors, Toll-like receptors, NF-κB, and MAPK signaling pathways, jointly regulating the function of macrophages (Gallenstein et al., 2023).

### 3.4 The role of the Notch signaling pathway in myocardial remodeling

#### 3.4.1 The association between Notch signaling pathway and myocardial remodeling

The Notch signaling pathway plays a crucial role in the occurrence and development of myocardial remodeling, and there is a close and complex association between Notch signaling pathway and myocardial remodeling. Myocardial remodeling is usually an adaptive response of the heart to various injurious factors, such as myocardial infarction and hypertension. In the early stage, it manifests as cardiac hypertrophy and interstitial fibrosis, and with the progression of the disease, it leads to a progressive deterioration of heart structure and function. Studies have shown that the Notch signaling pathway is activated in the initial stage of myocardial remodeling. For example, in animal models of myocardial remodeling induced by pressure overload, the expression of Notch1 and its ligands in myocardial tissue is significantly upregulated (Wei et al., 2024). Research indicates that activation of the Notch signaling pathway can promote the proliferation and differentiation of cardiomyocytes, but in a state of overactivation, it may trigger abnormal growth of cardiomyocytes, leading to cardiac hypertrophy (Zhang Q. et al., 2022). This hypertrophy is not only an adaptive response to cardiac pressure load but also an important component of myocardial remodeling.

Meanwhile, Notch signaling pathway also participates in the regulation of myocardial interstitial fibrosis. It can promote the proliferation and activation of fibroblasts, leading to the synthesis and secretion of more extracellular matrix components, such as collagen I and collagen III. The excessive deposition of these extracellular matrix components in the myocardial interstitium gradually forms fibrosis, destroying the normal structure and function of the myocardium and further promoting the development of myocardial remodeling (Bakalenko et al., 2024).

Furthermore, the sustained activation of Notch signaling pathway during the process of myocardial remodeling also affects the electrophysiological characteristics of the heart, leading to complications such as arrhythmias and further exacerbating the adverse consequences of myocardial remodeling. When myocardial remodeling occurs, the electrophysiological activity of the heart changes, and the Notch signaling pathway plays a regulatory role in this process. Some studies have found that abnormal activation of Notch signaling pathway interferes with the expression and function of ion channel proteins in cardiomyocytes following myocardial infarction (Onat et al., 2023). Therefore, gaining a deep understanding of the multi-dimensional association between Notch signaling pathway and myocardial remodeling is of great significance for revealing the pathogenesis of myocardial remodeling and developing effective treatment strategies.

#### 3.4.2 The prospect of the Notch signaling pathway as an intervention strategy for myocardial remodeling

The prospect of the Notch signaling pathway as an intervention strategy for myocardial remodeling is becoming increasingly clear, and its potential in the field of cardiac disease treatment is gradually being tapped. Studies have shown that by regulating the Notch signaling pathway, one can affect the survival, proliferation, and differentiation of myocardial cells, thereby intervening in the process of myocardial remodeling. Therefore, inhibiting Notch signaling pathway has become a potential therapeutic strategy aimed at reducing myocardial cell loss and slowing down the progression of cardiac remodeling.

In recent years, researchers have explored various methods to intervene in the Notch signaling pathway, including the use of Notch inhibitors, regulating the expression of key molecules in Notch signaling pathway, and altering interactions in the signaling pathway. One study pointed out that specific miRNAs such as miR-30e can affect the level of autophagy in myocardial cells by regulating the Notch1 signaling pathway, thereby alleviating ischemia-reperfusion injury. Additionally, the relationship between Notch signaling pathway and macrophage polarization is also considered a new perspective for intervening in myocardial remodeling (Li et al., 2022).

### 3.5 Crosstalk between Notch and other signaling pathways in the heart

The Notch signaling pathway does not function in isolation; the realization of its functions is highly dependent on intricate dialogue and integration with other key signaling pathways involved in cardiac development, homeostasis, and disease. This complex crosstalk network amplifies the regulatory dimensions of Notch signaling pathway, enabling it to coordinate processes ranging from early morphogenesis to late-stage pathological remodeling. A deep understanding of these interactions is paramount to comprehensively appreciating the role of Notch signaling pathway in the cardiovascular system.

#### 3.5.1 Interaction between Notch and Nodal signaling: from left-right asymmetry to cardiomyocyte aging

The interplay between Notch and Nodal signaling is established at the very early stages of cardiac development, collaboratively setting the foundation for heart left-right asymmetry. In the early embryo, Nodal signaling at the node establishes left-sided identity in the left lateral plate mesoderm through its downstream effector, Pitx2c. Research indicates that Notch signaling pathway acts as a right-side suppressor in this process. By inhibiting the expression of Nodal pathway-related genes in the right-sided heart region, Notch signaling pathway assists in establishing left-right axis asymmetry, which is a prerequisite for normal cardiac looping and chamber septation (De la Pompa and Epstein, 2012).

In recent years, this ancient developmental dialogue has been found to be recruited during aging in the adult heart. In the aging heart, Notch signaling pathway activity is generally declined, while some embryonic signals, including members related to the Nodal pathway, become aberrantly reactivated. Studies suggest that the attenuation of Notch signaling pathway may lead to a loss of its inhibitory control over these embryonic pathways. This can result in abnormal attempts at cell cycle re-entry by cardiomyocytes, metabolic disturbances, and the generation of a senescence-associated secretory phenotype, collectively accelerating the decline of cardiac function (Camacho-Encina et al., 2024). Therefore, the continuous role of the Notch-Nodal axis from development to aging illustrates the “repurposing” of signaling pathways across different stages of life.

#### 3.5.2 Synergy and antagonism with Wnt, BMP, and hippo pathways: regulating morphogenesis, regeneration, and disease

The Notch signaling pathway, together with the Wnt/β-catenin, BMP, and Hippo pathways, constitutes a core regulatory module for cardiac development and repair, characterized by dynamic and highly context-dependent interactions among them. The relationship between Notch and the Wnt/β-catenin signaling pathway is particularly complex, exhibiting both synergistic and antagonistic duality. For instance, during the early stages of cardiac precursor cell proliferation and ventricular cardiomyocyte differentiation, Notch signaling pathway can maintain the progenitor cell pool by inhibiting the Wnt/β-catenin pathway (Wang et al., 2013). Conversely, in the repair process following cardiac injury, moderate activation of Wnt signaling can promote the reactivation of Notch signaling pathway in the endocardium, which in turn stimulates cardiomyocyte proliferation by inducing paracrine factors such as BMP10 (Horitani and Shiojima, 2024). However, chronic and excessive activation of Wnt signaling can synergize with Notch signaling pathway to drive pathological fibrosis and myocardial remodeling.

The interplay between Notch and BMP signaling pathway serves as a classic paradigm in ventricular development and trabeculation. Specifically, Notch signaling pathway derived from the endocardium directly regulates the expression of BMP10, which acts as a crucial paracrine factor on cardiomyocytes, promoting their proliferation and differentiation. This Notch-BMP10 axis is indispensable for the normal thickening of the ventricular wall and the establishment of the trabecular network (Grego-Bessa et al., 2007). Furthermore, during valve development, BMP signaling cooperates with Notch signaling pathway to finely regulate the epithelial-mesenchymal transition (EMT) of endocardial cushion cells.

Additionally, an important link exists between Notch and the Hippo pathway. The core effector of the Hippo pathway, YAP/TAZ, is a key regulator of cell proliferation and tissue growth. Studies have found that in cardiomyocytes, the activated Notch intracellular domain (NICD) can physically interact with YAP, sequestering it in the cytoplasm and thereby inhibiting its pro-proliferative and pro-survival transcriptional activity (Wang J. et al., 2025). This mechanism provides a novel molecular explanation for how Notch signaling pathway suppresses excessive proliferation and maintains the terminally differentiated state in adult cardiomyocytes. In pathological models such as pressure overload, downregulation of Notch signaling pathway may lead to the loss of this inhibition on YAP, thereby contributing to the development of pathological cardiac hypertrophy.

#### 3.5.3 The emerging role of Notch in metabolic reprogramming and cardiomyocyte senescence

Cardiac energy metabolic homeostasis is central to maintaining its function. Recent studies indicate that Notch signaling pathway is directly involved in the metabolic regulation of cardiomyocytes. Notch signaling pathway activity can influence the metabolic substrate preference of cardiomyocytes; for example, inhibition of Notch signaling pathway may promote a shift from fatty acid oxidation to glucose metabolism, which is a common feature in the metabolic remodeling of heart failure (Gibb and Hill, 2018). Furthermore, Notch signaling pathway is closely linked to mitochondrial function. The Notch intracellular domain (NICD) can translocate to mitochondria, affecting mitochondrial dynamics (fusion/fission) and respiratory chain function. Dysregulation of this process may lead to excessive production of reactive oxygen species (ROS), accelerating cardiomyocyte senescence and apoptosis (Zhao Q. C. et al., 2019). In the aging heart, the widespread silencing of the Notch signaling pathway is associated with mitochondrial dysfunction and the accumulation of cellular senescence markers, suggesting that restoring Notch activity could be a potential anti-cardiac aging strategy.

#### 3.5.4 Hierarchical regulation of the Notch signaling pathway by non-coding RNAs and therapeutic potential

The Notch signaling pathway is finely regulated by a network of non-coding RNAs, including microRNAs (miRNAs) and long non-coding RNAs (lncRNAs), providing novel entry points for intervening in Notch signaling pathway.

Multiple miRNAs have been confirmed to directly target components of the Notch signaling pathway, forming feedback regulatory loops. For instance, the miR-106a-363 microRNA cluster carried by extracellular vesicles (EVs) promotes cardiomyocyte regeneration by targeting and suppressing key genes in the Notch3 signaling pathway (such as Jag1 and Hes1), thereby relieving the inhibition of the cardiomyocyte cycle. This mechanism provides new molecular targets for the treatment of ischemic heart disease (Jung et al., 2021).

These regulatory relationships endow non-coding RNAs with potential as therapeutic tools. Through extensive crosstalk with other core signaling pathways, metabolic networks, and the regulatory layer of non-coding RNAs, the Notch signaling pathway constitutes a multidimensional and dynamic regulatory system. It is precisely these complex interactions that determine the specificity and diversity of Notch signaling pathway functions within specific spatiotemporal contexts. Future therapeutic strategies targeting the Notch signaling pathway in cardiovascular diseases must fully consider its specific “interactome” background, which varies by cell type and pathological stage, to achieve precise intervention.

## 4 Research methods and technological advances

### 4.1 Research models of the Notch signaling pathway

In exploring the complex mechanisms of the Notch signaling pathway, researchers rely on a variety of model systems to elucidate its role in cardiac development, disease, and regeneration. Cell line models, such as H9c2 cardiomyocytes, are among the preferred tools due to their ease of manipulation and the controllability of culture conditions. By introducing activators or inhibitors of Notch signaling pathway, such as γ-secretase inhibitors, into these cells, researchers are able to observe the effects of Notch signaling pathway changes on cell proliferation, apoptosis, and function (Theis et al., 2015). Such experiments have revealed the key role of Notch signaling pathway in the survival and functional regulation of cardiomyocytes, providing a foundation for understanding heart diseases. Animal models, especially transgenic and knockout mice, offer the opportunity to study the Notch signaling pathway in an *in vivo* environment.

Additionally, disease models, such as acute lung injury and sepsis in rat models, are used to investigate the dynamic changes of Notch signaling pathway under specific pathological conditions. In these models, the expression changes of Notch receptors and their downstream target gene Hes-1 are closely related to the disease progression, suggesting the potential of Notch signaling pathway as a diagnostic biomarker and therapeutic target for diseases.

### 4.2 The application of molecular biology techniques in Notch signaling pathway research

The application of molecular biology techniques in Notch signaling pathway research constitutes a key tool for understanding this complex signaling pathway. Gene knockout technologies, especially the CRISPR-Cas9 system, have become powerful means for exploring the functions of specific genes within the Notch signaling pathway (Wu et al., 2023). By precisely deleting or inserting specific sequences, scientists are able to observe the impact of the absence or overexpression of various components in the Notch signaling pathway on cellular behavior, thereby revealing the specific roles of Notch signaling pathway in heart development, disease occurrence, and progression.

Protein interaction studies are an indispensable part of Notch signaling pathway research. The yeast two-hybrid system and co-immunoprecipitation techniques are used to identify interactions between Notch receptors and their ligands, as well as the patterns of recognition among key proteins in the Notch signaling pathway (Docshin et al., 2024). These techniques not only help to understand the activation mechanism of Notch signaling pathway but also promote in-depth exploration of the role of Notch signaling pathway in intercellular communication.

High-throughput sequencing technologies, particularly RNA sequencing, have become important tools for assessing the downstream gene expression profiles regulated by Notch signaling pathway. By comparing the transcriptomes of samples before and after Notch signaling pathway activation, researchers are able to identify key gene sets regulated by Notch signaling pathway, thereby revealing the molecular mechanisms of Notch signaling pathway in heart development, congenital heart disease, and myocardial regeneration processes (Rammah et al., 2022).

### 4.3 The latest advances in animal experiments and preclinical research

Animal experiments play a crucial role in exploring the relationship between the Notch signaling pathway and heart diseases. Recent studies have focused on how the Notch signaling pathway affects the repair mechanisms following myocardial infarction. By constructing mouse models of myocardial infarction, scientists have observed that changes in Notch signaling pathway activity are closely associated with myocardial cell survival, angiogenesis, and the recovery of heart function. Preclinical research has further expanded the therapeutic application prospects of the Notch signaling pathway in heart diseases. For example, the use of adeno-associated virus (AAV) vectors to deliver RBP-J short hairpin RNA (shRNA) can specifically inhibit Notch signaling pathway, thereby assessing its impact on mouse models of myocardial infarction. Such studies not only validate the mechanism of action of the Notch signaling pathway in heart diseases but also provide a theoretical basis for the development of new therapeutic strategies (Zhou et al., 2015).

Additionally, animal experiments have revealed the connection between the Notch signaling pathway and congenital heart diseases. By observing mice carrying mutations in Notch receptors and ligands, researchers have found that these mutations can lead to a series of cardiac developmental abnormalities, including structural defects and functional disorders of the heart (Sun, 2009).

### 4.4 Technical challenges and future directions in Notch signaling pathway research

The technical challenges and future directions in Notch signaling pathway research are intertwined with complexity and opportunities. Currently, the widespread expression of the Notch signaling pathway across various tissues presents a significant obstacle to the development of heart-specific drugs, as these medications may trigger systemic adverse reactions. This challenge necessitates that researchers consider the tissue-specific nature of the Notch signaling pathway and its intricate interactions with other signaling pathways when designing therapeutic strategies.

In the process of exploring the mechanisms of the Notch signaling pathway, scientists also face another technical challenge: how to accurately resolve the dynamic changes and functional diversity of Notch signaling pathway under a variety of cellular environments and pathophysiological conditions. Existing studies have shown that the role of the Notch signaling pathway in cardiac development and disease is not static but changes with environmental and temporal variations (Xu et al., 2024). Therefore, the development of high-throughput screening techniques and *in vivo* imaging technologies to monitor the activity of Notch signaling pathway in real-time under different conditions will be crucial for future research.

In the face of these challenges, interdisciplinary collaboration has become a powerful driving force in advancing Notch signaling pathway research. By integrating knowledge and techniques from fields such as bioinformatics, materials science, nanotechnology, and clinical medicine, researchers are able to examine research questions related to the Notch signaling pathway from a broader perspective and propose innovative solutions.

## 5 Research trends and future prospects

### 5.1 New perspectives in Notch signaling pathway research

In recent years, new perspectives on Notch signaling pathway research have emerged, driving the field of cardiac diseases forward. Emerging studies are beginning to explore the new roles of the Notch signaling pathway in the repair of myocardial ischemic injury. This shift not only reveals the complex mechanisms of Notch signaling pathway in the repair of myocardial ischemic injury but also suggests that the Notch signaling pathway may serve as a new target for clinical treatment of myocardial ischemia.

The Notch signaling pathway plays a more refined role in the recovery process following myocardial infarction. The regulatory mechanisms of the Notch3 receptor and its role in congenital coronary microvascular dysfunction, pericyte/progenitor cell recruitment, and microvascular maturation disorders provide new insights into the recovery of heart function after myocardial infarction.

To overcome these challenges, researchers are developing new tools and technologies to gain a deeper understanding of the role of the Notch signaling pathway in cardiovascular diseases. For example, Gamma-secretase inhibitors (GSIs), a class of inhibitors that have entered clinical development, have shown potential in the field of oncology (Ren et al., 2021).

### 5.2 The potential application of Notch signaling pathway in the treatment of heart diseases

The potential application of the Notch signaling pathway in the treatment of heart diseases is increasingly receiving attention, opening up new avenues for the intervention of cardiovascular diseases. Given the key role of Notch signaling pathway in cardiac development, myocardial regeneration, and the formation of congenital heart diseases, it shows great potential as a therapeutic target. However, the complexity of the Notch signaling pathway means that its regulation is not straightforward. The development of specific regulatory drugs is challenging due to the widespread expression of Notch signaling pathway in various tissues, which could lead to toxic side effects (Zhou et al., 2022). Additionally, the intertwining of the Notch signaling pathway with other signaling networks complicates targeted therapy. Nevertheless, researchers are exploring various strategies, including small molecule inhibitors, antibodies, and peptide substances, in an effort to achieve precise regulation of Notch signaling pathway (Aggarwal, 2021). These efforts are aimed at developing therapies that can effectively intervene in Notch signaling pathway without disrupting other important physiological processes.

MicroRNAs (miRNAs), as important regulators of the Notch signaling pathway, play a crucial role in the occurrence and development of cardiovascular diseases. miRNAs can regulate angiogenesis and the fate determination of cardiomyocytes by targeting key molecules in the Notch signaling pathway (Hashemi et al., 2022). Intervention targeting miRNAs and the Notch signaling pathway may become a new direction for personalized medicine in cardiovascular diseases, offering more precise and individualized treatment plans for patients.

### 5.3 The role of interdisciplinary collaboration in Notch signaling pathway research

The role of interdisciplinary collaboration in Notch signaling pathway research has become increasingly prominent, promoting the development and deepening of the field. Biological research provides the foundational knowledge of the Notch signaling pathway, elucidating its key role in determining cell fate. Medical research focuses on the association between Notch signaling pathway abnormalities and diseases such as congenital heart disease, exploring its potential as a therapeutic target. Materials scientists develop new biomaterials, providing platforms for myocardial regeneration research, while engineers design precision instruments to enhance the accuracy and efficiency of Notch signaling pathway research.

### 5.4 Long-term goals of Notch signaling pathway research

Setting long-term goals is crucial for Notch signaling pathway research, aiming to gain a deeper understanding of the precise regulatory mechanisms of this pathway in the cardiovascular system. Researchers are dedicated to exploring how Notch signaling pathway precisely regulates each stage of heart development, from heart formation, differentiation to growth, with each step being a focus of research. With technological advancements, the role of the Notch signaling pathway in myocardial regeneration and remodeling has become a research hotspot (Gao et al., 2021). The goal is to elucidate how Notch signaling pathway affects myocardial cell apoptosis, autophagy, and interaction with macrophages, thereby promoting myocardial regeneration. By regulating Notch signaling pathway, researchers aim to develop new therapies that promote myocardial repair and remodeling, bringing benefits to patients with heart diseases.

As a classic signaling pathway, the Notch signaling pathway plays an important role in regulating cellular metabolism, differentiation, proliferation, and apoptosis. Recent studies have found that the Notch signaling pathway is involved in the regulation of metabolic reprogramming in both tumor and non-tumor cells, and there has not been an article summarizing this to date (MacGrogan et al., 2018). In the long term, the ultimate goal of Notch signaling pathway research is to achieve personalized medicine. By deeply investigating the mechanism of Notch signaling pathway in individual differences, scientists strive to find the best treatment plans tailored to the characteristics of different patients. Moreover, interdisciplinary collaboration plays an increasingly important role in Notch signaling pathway research. The integration of fields such as bioinformatics, genetics, and cell biology will accelerate the progress of Notch signaling pathway research, opening up new paths for the prevention and treatment of heart diseases (Minnis-Lyons et al., 2021).
